# Were policies in Brazil effective to reducing *trans* fat from industrial origin in foods?

**DOI:** 10.11606/S1518-8787.2018052000292

**Published:** 2018-03-14

**Authors:** Flávia da Silva Lima Dias, Mário Ferreira Lima, Patricia Coelho de Velasco, Rosana Salles-Costa, Fátima Lúcia de Carvalho Sardinha, Maria das Graças Tavares do Carmo

**Affiliations:** I Universidade Federal do Rio de Janeiro. Instituto de Nutrição Josué de Castro. Laboratório de Bioquímica Nutricional. Rio de Janeiro, RJ, Brasil; II Universidade Federal do Rio de Janeiro. Instituto de Nutrição Josué de Castro. Departamento de Nutrição Social e Aplicada. Rio de Janeiro, RJ, Brasil

**Keywords:** Industrialized Foods, *Trans* Fatty Acids, analysis, Food Labeling, legislation & jurisprudence, Legislation, Food

## Abstract

**OBJECTIVE:**

To determine the *trans* fatty acids content of processed foods frequently consumed by adults living in a Rio de Janeiro, Brazil, after the enactment of a mandatory *trans* fatty acids labelling policy.

**METHODS:**

Between February 2014 and January 2015, a specifically dietary questionnaire was completed by 107 adults to assess the frequency of processed foods consumption. The most commonly consumed products from the survey, including vegetable oils, margarine, biscuits, snacks, cheese bread (*pão de queijo*), french fries, cheeseburger and ice cream, were then analyzed for their *trans* fatty acids content using gas chromatography with a flame ionization detector.

**RESULTS:**

Differences in the levels of *trans* fatty acids were observed among 22 products analyzed, considering that *trans* fatty acids content ranged between 0.0 g/100 g in samples of cream cracker biscuit 1 and olive oil to 0.83 g/100 g in samples of cheeseburger (fast food), 0.51 g/100 g in samples of frozen *pão de queijo* and 12.92 g/100 g in samples of chocolate sandwich cookies with cream filling 2. The overall *trans* fatty acids content of the different samples of margarine brands was 0.20 g/100 g for brand 1 and 0.0 g/100 g for brand 2. These data are significantly lower than those observed in a survey conducted in 2003, when the regulation had been enacted.

**CONCLUSIONS:**

Our data indicate that Brazilian regulation is very likely implicated in the observed drop in *trans* fatty acids of the most processed foods but has yet to eliminate them, which reinforces the urgent need to revise the legislation, since a minimum amount of *trans* fat does not mean that the food product does not contain this type of fat.

## INTRODUCTION

*Trans* fatty acids (TFA) refer to a group of unsaturated fatty acids that contain one or more double bonds in a *tran*s geometric configuration^17^. They are present in meat and dairy products as a by-product of fermentation in ruminant animals and in vegetable fats because of partial hydrogenation of the oils containing polyunsaturated fats^11^. The consumption of naturally occurring TFA originated from ruminants by human beings is generally low and it has been suggested – based on evidence – that it does not have adverse effects on health^17^ ^,^ ^32^. On the other hand, there is evidence that the consumption of industrially produced *trans*-fatty acids is associated with an increased risk of cardiovascular disease^16^ ^,^ ^24^ ^,^ ^25^, type 2 diabetes mellitus, Alzheimer’s disease, cancer, as well as several other diseases^11^ ^,^ ^14^ ^,^ ^24^ ^,^ ^25^. A TFA intake of 5 g per day is associated with a 25% increase in the risk of ischemic heart disease^30^. As a consequence, the elimination from the food supply of partially hydrogenated vegetable oils containing TFA derived from industrial processes has been considered a very efficient action in the public health area in order to improve population diet and reduce the risk of chronic diseases^9^ ^,^ ^22^.

Hydrogenation of vegetable oils has been done commercially in Brazil since the late 1950s, as part of the industrial production process of shortening and hard margarine. This process led to a quick replacement of animal fats with processed vegetable fats in the diet of the Brazilian population. During this partial hydrogenation process, the temperature of polyunsaturated oils is increased in the presence of nickel or other catalyst metals and the material is exposed to hydrogen gas. Some of the double bonds are saturated in this process while others undergo geometric isomerism, changing to a *trans* configuration, or positional isomerism, being shifted to a different position in the aliphatic chain.

Over the last decades, the production of partially hydrogenated vegetable oils increased in Brazil. Because of their low cost, long shelf life, oxidative stability, semi-solidity at room temperature, and suitability for commercial frying^17^, these fats have been widely used in the production of several foods, such as margarine, chocolate spreads, biscuits, potato chips, and bakery products^1^. A study conducted in Brazil in 2003 showed that the average TFA content in fried potatoes from fast food restaurants was 4.74 g/100 g; in ice cream, the values varied from 0.041 g/100 g to 1.41 g/100 g; and in cookies, the values varied from 2.81 g/100 g to 5.60 g/100 g^5^.

The food industry has increased efforts to reduce TFA amounts, especially in processed foods, due to the damaging health effects of TFA intake^23^. Most public health agencies have implemented labelling or ingredient restrictions on *trans* fats, including Brazil. In 2003, the National Sanitary Surveillance Agency (ANVISA) made obligatory the declaration of the TFA quantity on the nutritional labels of processed food commercialized in Brazil^12^. In addition, according to The Executive Board Resolution (*Resolução da Diretoria Colegiada*) no. 359 and no. 360, processed foods that contain an amount of *trans* fat less than or equal to 0.2 g/portion can be considered as “does not contain *trans* fat” and was also described as “not significant” in the Resolution^21^. Moreover, when the quantity of *trans* fat does not reach the minimum limit recommended by the legislation (0.2 g/portion), the food industry is under no obligation to display the content on the label, thus impairing any nutritional analysis on food regarding *trans* fat^28^.

It is necessary to keep active the process of *trans* fat content assessment in food labels available in Brazil in order to estimate intakes and to support the choice of public policies related to risk management. This study aims to provide updated information on the levels of TFA found in processed foods with high level of consumption in Rio de Janeiro, Brazil, after the TFA labelling became mandatory by legislation. To assess the changes in *trans* fat levels in Brazilian industrialized foods over the past decade, the data are compared with surveys done in 2003, which evaluated manufactured foods^5^.

## METHODS

### Study Design and Participants

The survey carried out in 2014–2015 included 107 adults, 20 to 50 year old male and female living in Duque de Caxias, state of Rio de Janeiro, Brazil. Duque de Caxias city was chosen for the study because it is an important industrial center with a population of about 842,686, located in the metropolitan area of Rio de Janeiro and within a distance of no more than 30 km from Rio de Janeiro city^6^. Considering it is an exploratory study, the sample was formed by convenience. Participants were recruited from a basic public health unit. The Human Ethics Committee of the Universidade Federal do Rio de Janeiro approved the study and all participants gave informed consent. Participants were excluded if they were affected by a metabolic disorder or were taking medication known to alter plasma lipids.

The self-administered questionnaire on the frequency of processed foods consumption was applied. The questionnaire inquired only about the frequency of consumption without specifying portion size. Participants were asked how often, on average, they had consumed these foods over the past six mo. Six predefined frequency categories ranging from “never/seldom” to “four or more times per week” were used. Also, open questions were conducted about daily consumption of five different types of margarine and oil. Foods with a frequency of consumption reported to be equal to or higher than two to four times a week were considered the most commonly consumed ones. Twenty-two products were selected for analysis (Box).

**Box:** Products selected for analysis of *trans* fatty acids.

Food category	Selected food
Oils and fats	Soybean oil brand 1 Soybean oil brand 2 Olive oil Margarine brand 1 Margarine brand 2
Cream crackers^a^ and cookies	Cream crackers brand 1 Cream crackers brand 2 Cookies without filling Chocolate sandwich cookies with chocolate-flavored filling brand 1 Chocolate sandwich cookies with chocolate-flavored filling brand 2 Plain sandwich cookies with chocolate-flavored filling
Snacks	Ham-flavored Barbecue-flavored Cheese-flavored brand 1 Cheese-flavored brand 2 Shoestring potatoes brand 1 Shoestring potatoes brand 2
Fast foods	Cheeseburger French fries
Frozen food	*Pão de queijo* ^b^ Ice cream brand 1 - chocolate, vanilla, and strawberry flavors Ice cream brand 2 - chocolate, vanilla, and strawberry flavors

^a^ Crackers made from water, flour, and fat.

^b^ A savory bun made from sour manioc starch, eggs, milk, oil, and cheese.

### Sampling

The analysis was performed on samples of food purchased by the participants in supermarkets or other usual points of sale listed. All samples were stored following the recommendations found on their labels and the ready-to-eat takeaway products were stored in the refrigerator and frozen as quickly as possible. If certain sub-samples required any kind of preparation or cooking, they were processed strictly following the manufacturers’ instructions, by usual domestic practices.

For each product, five samples from three different batches were purchased. To allow for thorough homogenization of samples and to obtain representative aliquots^26^ of the products analyzed, five samples from the same batch were homogenized to obtain a composite sample. The same was done for the other two batches purchased. Three composite samples (each with 15 sub-samples) were formed and prepared for TFA analysis. A total of 42 samples were analyzed.

### Sample Preparation

Sub-samples were homogenized and combined into composite samples for analysis on an equal weight basis and were stored frozen at -40^o^C until required for analysis.

The homogenization of the samples was performed at room temperature. Solid samples were homogenized in 0.9% saline solution using a mini processor, while liquid and creamy samples, such as oils and margarine, were shaken in glass tubes without processor usage.

### Sample Analysis

#### Lipid extraction and preparation of fatty acid methyl esters (FAME)

The extraction of lipids, saponification, and methylation of fatty acids contained in the samples were performed using the method of the Association of Official Agricultural Chemists Ce 1j-07^2^ and with FAME derivatization procedure using BF3 14% in methanol according to International Standards – ISO 5509 (2000). The FAME in the fat samples were collected in vials proper for gas chromatograph (GC) autosampler and stored at -20ºC for subsequent analysis of fatty acids.

#### Gas Chromatograph Analysis of Fatty Acid Methyl Esters

The FAME were identified by the Agilent model 7890 GC instrument (Palo Alto, CA, USA) equipped with a flame ionization detector and the EZ Chrom Elite software and using the modified temperature program^8^ ^,^ ^19^. A highly polar fused silica capillary column SP^TM^-2560 0.25 mm i.d. × 100 m length, coated with 100% cyanopropyl polysiloxane stationary phase, film thickness 0.20 μm, were used (Supelco, Inc., Bellefonte, PA, USA) to separate the FAME. The temperature of injector and detector were maintained at 250.0°C, with a split ratio of 1:100, 1 μL of standard or sample, equivalent to 20 μg of total FAME were injected using an autosampler device in each GLC run. The oven temperature program developed was: initial temperature 100.0°C ramped 3.0°C/min – 140.0°C, ramped 0.5°C/min - 170°C, ramped 4.0°C/min – 220.0°C, maintained for 35 minutes. Carrier gas hydrogen, make up gas nitrogen, and hydrogen and air for the flame ionization detector were used. Each determination was run at least in triplicate. The individual FAME was achieved by comparison of its relative retention time to those of commercial standards (GLC-463- Nu-Chek Prep. Inc) and other referenced FAME standards. Quantification of the fatty acids was carried out by calculating the response factors of individual fatty acid isomers. Total TFA was calculated as the sum of t7-C18:1(*trans*-7-octadecenoic acid), t9-C18:1(*trans* elaidic acid), t11-C18:1(*trans* vaccenic acid), t12-C18:1(*trans*-12-octadecenoic acid) and t9, t12-C18:2 (*trans* linolelaidic acid).

## Statistical Analysis

The results are expressed as mean value and standard deviation. The results were compared using analysis of variance with a 5% significance level^29^. For all table analysis and preparation we used Microsoft® Office Excel (version 10.0).

## RESULTS AND DISCUSSION

The main findings of this cross-sectional study show that there was a drop in TFA content of processed foods frequently consumed in Rio de Janeiro, Brazil, that coincided with labelling regulation employed in 2003 by the ANVISA. Although significant progress has been made with labelling regulation in Brazil, TFA levels need to be reduced even more, particularly in fast foods and some types of biscuits.

The TFA comprised isomers of 16:1, 18:1 and 18:2 acids and *trans* 18:1 isomers were the major group of TFA present in most brands analyzed, ranging from 0.0 to 12.38 g/100 g of product (Table). Highest TFA concentrations were observed in brand 2 cream-filled chocolate sandwich cookies while no TFA were present in olive oil and brand 1 cream cracker biscuit. Unlike our results, a previous study^18^ that also evaluated cream cracker biscuits in Brazil revealed TFA levels ranging from 12.2% to 31.2% of total fatty acids and the mean value was 20.1%. However, that study was conducted before the change in Brazilian legislation^12^ that demanded TFA labeling on all packaged foods. Dias and Gonçalves^7^ assessed the nutritional labeling of 150 samples of different brands of biscuits, chocolates, and ice cream. Those authors found that about 55% of the products analyzed were not adequate to the ANVISA’s legislation, particularly regarding the portion size information and the TFA content. In another study, the TFA level in some margarines purchased at supermarkets in Brazil remained over 50% after regulation^3^. Finally, when comparing the TFA concentrations found in this study with those found by Winter et al.^33^, there is a high reduction of this type of fatty acid in straw potatoes − also known as shoestring fries potatoes − sold in Brazil (almost 15 g/100 g), probably by replacing the hydrogenated vegetable fat used for frying with other types of oils. Therefore, the removal of partially hydrogenated vegetable fat, containing industrially produced TFA, in processed food by Brazilian industries was not an easy and fast procedure when compared to other countries.

**Table:** *Trans* fatty acid (TFA) content (g/100 g) (presented by mean and SD) of the analyzed samples.

Product	C16:0	C18:2 n6	C16:1 t^a^	C18:1 t^b^	C18:2 t^c^	Total SFA	Total TFA	TFA per serving
Soybean oil brand 1	10.97 (3.33)	48.26 (5.36)	0.00	0.00	0.09 (0.01)	14.74	0.09	0.01
Soybean oil brand 2	10.02 (0.62)	47.56 (3.67)	0.00	0.00	0.26 (0.01)	13.44	0.26	0.03
Olive oil	9.30 (0.55)	4.90 (0.93)	0.00	0.00	0.00	12.62	0.00	0.00
Margarine brand 1	10.99 (1.22)	39.08 (3.95)	0.03 (0.10)	0.21 (0.10)	0.65 (0.01)	16.87	0.86	0.09
Margarine brand 2	3.76 (1.28)	15.91 (7.88)	0.00	0.00	0.20 (0.07)	8.18	0.20	0.03
Cream crackers brand 1	7.11 (0.74)	2.61 (0.25)	0.00	0.00	0.00	8.35	0.00	0.00
Cream crackers brand 2	11.11 (1.89)	4.13 (0.80)	0.00	0.04 (0.01)	0.08 (0.02)	12.95	0.12	0.04
Cookies	6.94 (0.52)	2.61 (0.23)	0.00	0.00	0.01 (0.00)	8.28	0.01	0.00
Chocolate sandwich cookie with chocolate-flavored filling brand 1	8.27 (0.91)	7.29 (0.80)	0.00	0.47 (0.11)	0.05 (0.01)	11.44	0.52	0.16
Chocolate sandwich cookie with chocolate-flavored filling brand 2	2.47 (0.12)	1.52 (0.12)	0.00	12.38 (1.16)	0.54 (0.02)	4.71	12.92	3.88
Plain sandwich cookie with chocolate-flavored filling	4.41 (0.10)	6.69 (0.39)	0.00	0.06 (0.00)	0.13 (0.00)	6.97	0.19	0.00
Ham-flavored savory biscuit	11.00 (0.98)	3.74 (0.35)	0.00	0.00	0.06 (0.00)	12.78	0.06	0.02
Barbecue-flavored savory biscuit	3.02 (0.20)	14.35 (0.88)	0.00	0.00	0.05 (0.02)	4.46	0.05	0.01
Cheese-flavored savory biscuit brand 1	1.91 (0.17)	1.70 (0.53)	0.01 (0.00)	0.05 (0.01)	0.02 (0.00)	2.66	0.07	0.00
Cheese-flavored savory biscuit brand 2	5.97 (0.04)	1.88 (0.02)	0.01 (0.00)	0.00	0.04 (0.00)	7.12	0.05	0.00
Shoestring potatoes brand 1	15.00 (0.21)	4.32 (0.03)	0.02 (0.00)	0.08 (0.00)	0.11 (0.01)	17.81	0.21	0.05
Shoestring potatoes brand 2	6.63 (0.26)	15.42 (0.77)	0.01 (0.00)	0.05 (0.00)	0.21 (0.01)	7.89	0.27	0.05
Cheeseburger (fast food)	4.36 (0.16)	1.20 (0.09)	0.00	0.75 (0.04)	0.08 (0.02)	9.49	0.83	-^d^
French fries (fast food)	2.04 (0.24)	7.31 (0.69)	0.00	0.00	0.01 (0.00)	2.84	0.07	-^d^
Frozen *pão de queijo*	3.71 (0.42)	4.97 (0.44)	0.03 (0.01)	0.43 (0.01)	0.04 (0.01)	7.30	0.51	0.23
Ice cream 1	3.18 (0.03)	0.71 (0.05)	0.00	0.01 (0.00)	0.03 (0.00)	3.91	0.04	0.02
Ice cream 2	2.99 (0.14)	0.74 (0.03)	0.00	0.00	0.02 (0.00)	3.92	0.02	0.01

t: *trans*; c: *cis*

^a^ C16:1 9t, C16:1 11t

^b^ C18:1 6t+8t, C18:1 9t,C18:1 10t, C18:1 11t, C18:1 13t+14t

^c^ C18:2 9t.12t, C18:2 9t.12c, C18:2 11t.15c, C18:2 7t. 9t

^d^ Portion not established.

Recently we demonstrated low levels of TFA in biscuits frequently consumed by Brazilian college students in the period of 2009–2011^8^. In addition, high levels of palmitic acid were found, indicating the likely use of palm oil in manufacturing. Likewise, in the current study, palm oil appears to be the main hydrogenated vegetable fat substitute in the manufacture of processed foods, since high levels of saturated fatty acids, particularly palmitic acid, were found in the samples. Exceptions were brand 2 margarine and barbecue-flavored salty biscuit, in which soybean oil was possibly used due to the higher content of polyunsaturated fatty acids, particularly linoleic acid (Table). These results highlight another concern: the use of alternative fats to decrease TFA contents produces industrialized food products with high levels of saturated fatty acids or low content of essential fatty acids. Additionally, it has been reported that a high intake of palm oil contributes to the development of coronary heart disease^10^ ^,^ ^15^ ^,^ ^31^. Then, countries whose crops originate oils rich in mono- or polyunsaturated fatty acids^27^ may find it easier to change from partially hydrogenated vegetable oils to those with fewer adverse effects on health. In Argentina, for instance, the shift to high-oleic sunflower oil in order to reformulate food production was far easier than similar modifications that occurred in Brazil, where a significant increase in the palm oil production has taken place over the last decades^20^.

The use of palm oil as the main substitute for hydrogenated vegetable fat was also observed by Gagliardi et al.^13^ in foods sold in Brazil, such as margarines, cookies and fast food products. As ascertained in the present study, there was an increase in palmitic acid levels associated with the reduction of TFA in these foods.

There are alternatives to TFA that can be used in product reformulation, including interesterification. The process allows the production of *trans* fat free or very low content of TFA, from the rearrangement of fatty acids in ester bonds of glycerol and consequent change in melting point and crystallization of fat. As the result, this process influences the physical characteristics of fats. Interesterified fats for commercial use are usually generated from the random interesterification of a liquid oil (for example soy oil) with a fully hydrogenated fat or blends of palm-stearin fractions^4^. The final product presents the functionality desired by the food industry, with an increased proportion of solid fat content without TFA. This process could be the responsible for the low TFA content in brand 1 margarine.

According to Brazilian legislation, a product can be classified as TFA-free when it contains, per portion, less than 0.2 g^21^. In the present study, only frozen cheese bread and brand 2 cream-filled chocolate sandwich cookies can be considered, according to Brazilian legislation, a source of TFA products, with 0.23 g and 3.76 g per serving of the product. However, attention should be paid to the question of the amount consumed since often ingestion is greater than single portion, resulting in an intake of considerable amounts of TFA. Thus, those who consume higher quantities may represent a significant intake of *trans* fats. On the other hand, stricter regulation has been considered in recent years in the United States by the Food and Drug Administration (FDA 2013), where steps have been taken to no longer define partially hydrogenated oils – the primary source of industrially produced TFA – as generally recognized as safe. In 2015, the FDA has made its final determination that there is no longer a consensus among qualified experts that partially hydrogenated oils are generally recognized as safe for any use in human food. Thus, following the global trend towards the elimination of *trans* fat consumption, it would be important to revise the official Brazilian documents on *trans* fat.

In the present study, when different brands of the same product are compared, it is observed that the food industry may use different lipid sources to manufacture similar items. For example, some biscuits were made with partially hydrogenated fat, while others were made with olive oil. Consequently, this could affect not only the lipid quality of the final product but also the price to the consumer – in market research, it was found that, overall, the products that had higher *trans* fatty acid levels are the cheaper ones. These differences were detected in oils and margarines, but mostly in sweet biscuits with filling. It is important to notice that the brand 2 of this biscuit, which showed high levels of TFA, was a product cheaper than the similar one. Additionally, the lipid composition indicates the use of hydrogenated vegetable fat as an ingredient in its industrial process. In fact, the official Brazilian legislation recommends that consumers consult the list of product ingredients because indications of the absence of *trans* fat in the nutritional information cannot be considered safe. However, Silveira^29^ detected different forms of fat source of TFA representation on food labels as “partially hydrogenated oils”, “hydrogenated vegetable fat”, “hydrogenated vegetable oil”, among others. In addition, terms may be found as “fat”, “vegetable cream”, and “margarine”, which do not inform whether or not it has TFA. For the products analyzed, we found terms such as “vegetable fat”, “hydrogenated vegetable fat”, “margarine”, “soybean oil”, “interesterified vegetable fat”, and “vegetable oil interesterified”. This lack of standardization may confuse consumers as to the quality of fat added to the product.

It is probable that TFA food content in Brazil is declining due to the food industry’s progress in reducing the *trans* fat content in a wide range of products and due to more awareness of food choices by the consumers. Researchers analyzed products in the city of Rio de Janeiro in 2003, prior to legislation aimed at reducing the use of hydrogenated fats by the food industry, and they found high levels of TFA^5^. On the other hand, data from the current study indicate low levels of these fatty acids in similar products, as shown in Figure 1.

**Figure:**
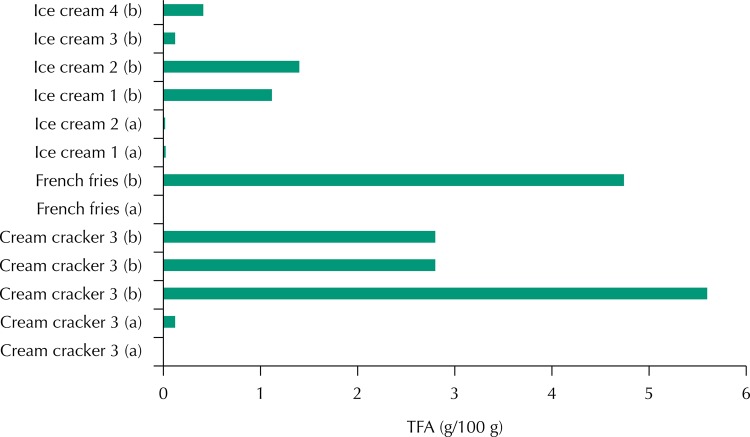
Comparison of *trans* fatty acid content (g/100 g) of cream crackers, french fries, and cheese bread in the present study (a) and values reported in the literature (5) before the introduction of Brazilian legislation on the subject (b). TFA: *trans* fatty acids

## CONCLUSIONS

TFA content varied between 0.00 g and 0.86 g/100 g with exception of one sample (12.92 g/100 g) and different values were found among similar products, indicating distinct lipid sources in food production.

The results found in this study and their comparison with studies conducted prior to the Brazilian legislation indicate that replacing TFA-rich fats with alternative sources has been done by the food industry, although it has not been observed in all analyzed products. However, there is little information about the quality of fats used in this replacement that could potentially result in increased consumption of saturated fats.
